# Operation for Perforated Gastric Ulcer

**Published:** 1900-12-01

**Authors:** 


### OPERATION FOR PERFORATED GASTRIC ULCER.

Dr. G. H. Hume,[1] of Newcastle-on-Tyne, publishes a series of eleven cases of perforated gastric ulcer on which he has operated, and like all other surgeons he urges that success depends largely upon early operation. In three cases the interval between the attack and the operation was from 43 to 48 hours, and all these cases died. In the cases that recovered the interval was ranged from 6 to 28 hours, and it is to be noted that in the 28-hour case the rupture took place when the stomach was empty, that absolutely nothing had been allowed by the mouth from the time of its occurrence, and that at the time of the operation so little extravasated fluid was met with that only slight sponging was required. On the other hand, one of the fatal cases was operated on only five and a quarter hours ofter the occurrence of perforation, and it may be that in this case the fatal issue was to be explained by the fact that shortly after the onset of the attack a large dose of castor oil had been administered by the mouth. A useful moral can be drawn from these facts.

[1] Lancet, Nov. 10.

